# Transcription Elongation and Tissue-Specific Somatic CAG Instability

**DOI:** 10.1371/journal.pgen.1003051

**Published:** 2012-11-29

**Authors:** Agathi-Vasiliki Goula, Agnieszka Stys, Jackson P. K. Chan, Yvon Trottier, Richard Festenstein, Karine Merienne

**Affiliations:** 1 Programme of Translational Medicine and Neurogenetics, Institute of Genetics and Molecular and Cellular Biology (IGBMC), UMR 7104-CNRS/INSERM/UdS, Illkirch, France; 2 Department of Medicine, Imperial College London, Hammersmith Hospital Campus, London, United Kingdom; The Hospital for Sick Children and University of Toronto, Canada

## Abstract

The expansion of CAG/CTG repeats is responsible for many diseases, including Huntington's disease (HD) and myotonic dystrophy 1. CAG/CTG expansions are unstable in selective somatic tissues, which accelerates disease progression. The mechanisms underlying repeat instability are complex, and it remains unclear whether chromatin structure and/or transcription contribute to somatic CAG/CTG instability *in vivo*. To address these issues, we investigated the relationship between CAG instability, chromatin structure, and transcription at the HD locus using the R6/1 and R6/2 HD transgenic mouse lines. These mice express a similar transgene, albeit integrated at a different site, and recapitulate HD tissue-specific instability. We show that instability rates are increased in R6/2 tissues as compared to R6/1 matched-samples. High transgene expression levels and chromatin accessibility correlated with the increased CAG instability of R6/2 mice. Transgene mRNA and H3K4 trimethylation at the HD locus were increased, whereas H3K9 dimethylation was reduced in R6/2 tissues relative to R6/1 matched-tissues. However, the levels of transgene expression and these specific histone marks were similar in the striatum and cerebellum, two tissues showing very different CAG instability levels, irrespective of mouse line. Interestingly, the levels of elongating RNA Pol II at the HD locus, but not the initiating form of RNA Pol II, were tissue-specific and correlated with CAG instability levels. Similarly, H3K36 trimethylation, a mark associated with transcription elongation, was specifically increased at the HD locus in the striatum and not in the cerebellum. Together, our data support the view that transcription modulates somatic CAG instability *in vivo*. More specifically, our results suggest for the first time that transcription elongation is regulated in a tissue-dependent manner, contributing to tissue-selective CAG instability.

## Author Summary

Several dominant genetic diseases, including Huntington's disease (HD) and myotonic dystrophy 1, are caused by the expansion of CAG/CTG repeats. These repeats are unstable in selective tissues. Repeat instability, resulting in the production of increasingly toxic mutant entities in the affected tissues, is proposed to accelerate disease progression. It is therefore essential to unravel the mechanisms contributing to tissue-selective somatic instability. *In vitro* and cell-based studies indicate that transcription is involved in CAG/CTG instability. However, the role of transcription in CAG/CTG instability *in vivo* has remained controversial, since mRNA tissue levels of CAG/CTG repeat-containing genes do not correlate with the tissue-specific pattern of instability. Moreover, it is unclear whether the transcriptional process would contribute *per se* to CAG/CTG instability or whether it is the increased chromatin accessibility associated with transcription that would promote instability. We addressed these issues using two HD transgenic mouse lines recapitulating HD tissue-specific instability. Our *in vivo* data indicate that high chromatin accessibility and transgene expression do not underlie tissue-selective CAG instability in HD, but suggest that the dynamics of transcription elongation is one mechanism contributing to this process.

## Introduction

The expansion of trinucleotide repeats (TNRs) is the causative mutation of more than fifteen neurodegenerative, neurological and neuromuscular genetic diseases, including Huntington's disease (HD), several dominant spinocerebellar ataxias (SCAs), myotonic dystrophy 1 (DM1), Friedreich ataxia (FRDA) and fragile X syndrome (FXS) [1]. TNR expansions become unstable and toxic above a threshold of 30 to 50 repeat units. TNR instability leads to repeat size variation, often biased towards further expansion, in the germline and in somatic tissues over time [2]–[4]. Remarkably, the toxicity and the instability of TNRs are tissue-selective in a disease-specific manner. In some diseases, including HD, DM1 and FRDA, the affected and ‘unstable’ tissues partially coincide, thereby accelerating disease progression [1], [5]–[7]. In HD, somatic instability of CAG repeats is most important in the striatum, a tissue that selectively degenerates [4], [6]. In DM1, somatic CTG instability is also most extensive in affected tissues, including the muscle and some brain regions [8]–[10]. Similarly, somatic GAA instability in FRDA is important in dorsal root ganglia and cerebellum, two tissues primarily affected in the disease [11], [12]. Thus, it is essential to decipher the mechanisms underlying tissue-selective instability of TNRs to better define the relationship between toxicity and instability.

CAG/CTG repeats as well as other TNRs form stable DNA secondary structures *in vitro*, and several studies support the view that the error-prone repair of these structures by overwhelmed DNA repair machineries contributes to TNR instability [1], [13]–[19]. Studies based on cell models also suggest that transcription is involved in CAG/CTG instability [20], [21] and formation of stable DNA:RNA hybrids (R-loops) at repeat sites might contribute to the process [22]–[24]. Furthermore, all TNR loci associated with diseases are located in transcribed regions of the genome, supporting the idea that transcription and TNR instability are linked. However, the role of transcription in TNR instability *in vivo* remains unclear [1]. First, if transcription contributes to TNR instability, one would expect that transcriptional activity at TNR genes correlates with tissue-selective repeat instability, which seems to be contradicted by previous studies [25]–[27]. For instance, Huntingtin (*HTT*) mRNA in the striatum of HD patients or rodent models was not increased when compared to tissues exhibiting minimal CAG instability, such as the cerebellum [25], [26]. Similarly, somatic CTG instability in selective tissues did not correlate with increased transcription levels in DM1 mice [27]. Second, it is unclear whether the transcriptional process would contribute *per se* to TNR instability or whether transcription would only facilitate access of transacting-factors such as DNA repair proteins, due to increased accessibility of chromatin at transcribed regions [28]. In the latter hypothesis, chromatin structure rather than transcriptional activity is expected to influence TNR instability.

It has been shown that TNRs, together with specific *cis*-elements, have the capacity to modify the chromatin environment by inducing a heterochromatinization process, eventually leading to transcriptional silencing such as in FRDA and FXS, which are caused by GAA and CGG expansions, respectively [29]–[31]. Thus, heterochromatin rather than accessible chromatin has been associated with unstable TNRs, pointing to an apparent paradox.

Here, we have investigated the relationship between chromatin structure, transcription and tissue-selective CAG instability by using R6/1 and R6/2 HD transgenic mouse lines. These two lines have been generated using the same HD transgenic construct, which consists of ≈1000 bp of the human *HTT* promoter, the entire *HTT* exon-1, including a CAG repeat expansion, and 262 bp of *HTT* intron-1 [32]. The transgene is integrated in different genomic loci, recently mapped to chromosome 3 and 4 in R6/1 and R6/2 lines, respectively [33], [34]. R6/1 and R6/2 mice develop a comparable HD-like phenotype. However, progression of the disease is much faster in R6/2 than in R6/1 mice, dying at ≈12 and ≈40 weeks, respectively. Importantly, somatic CAG instability in R6 mice exhibits a similar tissue-selectivity profile as in HD patients, indicating that the mechanisms underlying tissue selectivity are conserved between the two species [35]. Specifically, CAG instability in R6 lines and in humans is most marked in the striatum and minimal in the cerebellum. We previously showed that the level of somatic instability was higher in R6/2 than in R6/1 mice of similar ages, indicating that the instability rate might be increased in R6/2 mice [36]. CAG instability was also higher in R6/2 mice, irrespective of the repeat size (100 or 160 CAG repeats), than in R6/1 mice (130 CAG repeats) [36], suggesting that it is the chromatin context at the site of transgene integration and possibly the expression level of HD transgene, rather than the initial expansion size that underlies the different somatic instability levels.

To test the hypothesis that the chromatin structure and/or transcriptional activity at the HD locus might contribute to somatic CAG instability, we have performed chromatin immunoprecipitation experiments and expression analyses from the striatum and the cerebellum of R6/1 and R6/2 mice. Our results indicate that the HD transgene is integrated in a more euchromatinized and transcriptionally active region in R6/2 mice than in R6/1 mice, supporting the view that an active chromatin structure results in increased CAG instability. However, chromatin accessibility and transcription initiation at the HD locus were similar in the striatum and cerebellum of R6 mice, indicating that tissue-selective CAG instability is unlikely to be simply explained by an accessible or transcriptionally active chromatin state. Strikingly, H3K36 trimethylation and elongating RNA Pol II were increased at the HD locus in the striatum of both R6/1 and R6/2 mice. Together, our *in vivo* data suggest that transcription elongation mediates tissue-specific somatic CAG instability in HD.

## Results

### CAG instability rates in striatum and cerebellum of R6/1 and R6/2 mice

**Figure 1:**
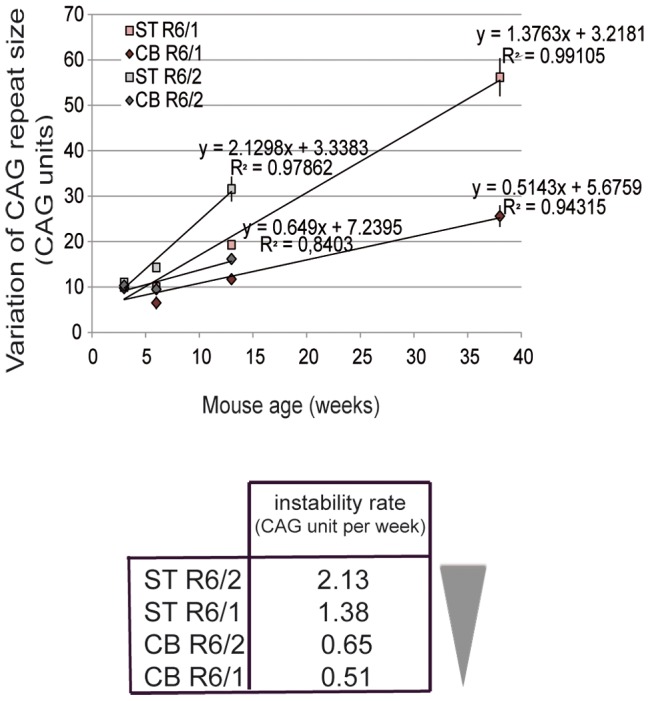
Somatic CAG instability rates in R6/1 and R6/2 striatum and cerebellum. Upper panel. The variation of the number of CAG repeats, a mean to assess instability, was measured over time in this study and previously [36]. The striatum and cerebellum of R6/1 and R6/2 mice were analyzed at 3 weeks, 6 weeks and 12 weeks and in addition at 38 weeks for R6/1 mice. R6/2 mice with 160 CAG repeats and R6/1 mice with 130 CAG repeats were analyzed. Error bars, sem. Instability increased more rapidly in R6/2 tissues relative to R6/1 tissues and in striatum as compared to cerebellum. Instability rates for the striatum and cerebellum of R6/1 and R6/2 mice were deduced from regression analyses, and correspond to regression coefficients. Lower panel. Ranking of instability rates according to mouse line and tissue.

To correlate the chromatin state and the transcriptional activity at the HD locus with the level of CAG instability in R6/1 and R6/2 striatum and cerebellum, we determined CAG instability rates. The level of instability of CAG repeats in mouse striatum and cerebellum was assessed in this study and in previous work [36]. R6/1 mice with 130 repeats were analyzed at 3, 6, 12 and 38 weeks and R6/2 mice with 160 repeats at 3, 6 and 12 weeks (R6/1 and R6/2 mice die around 40 and 12 weeks, respectively). To determine instability rates, we represented the level of CAG instability in R6/1 and R6/2 striatum and cerebellum, as measured by the variation of CAG repeat size, according to age (Figure 1). Interestingly, instability rates over time could be modeled using linear regression analyses (Figure 1). CAG instability rates, as measured by the regression coefficient (slope), ranked as follows: R6/2 striatum>R6/1 striatum>R6/2 cerebellum>R6/1 cerebellum (Figure 1).

### Chromatin at the HD locus is less accessible in R6/1 tissues compared to R6/2 tissues

**Figure 2:**
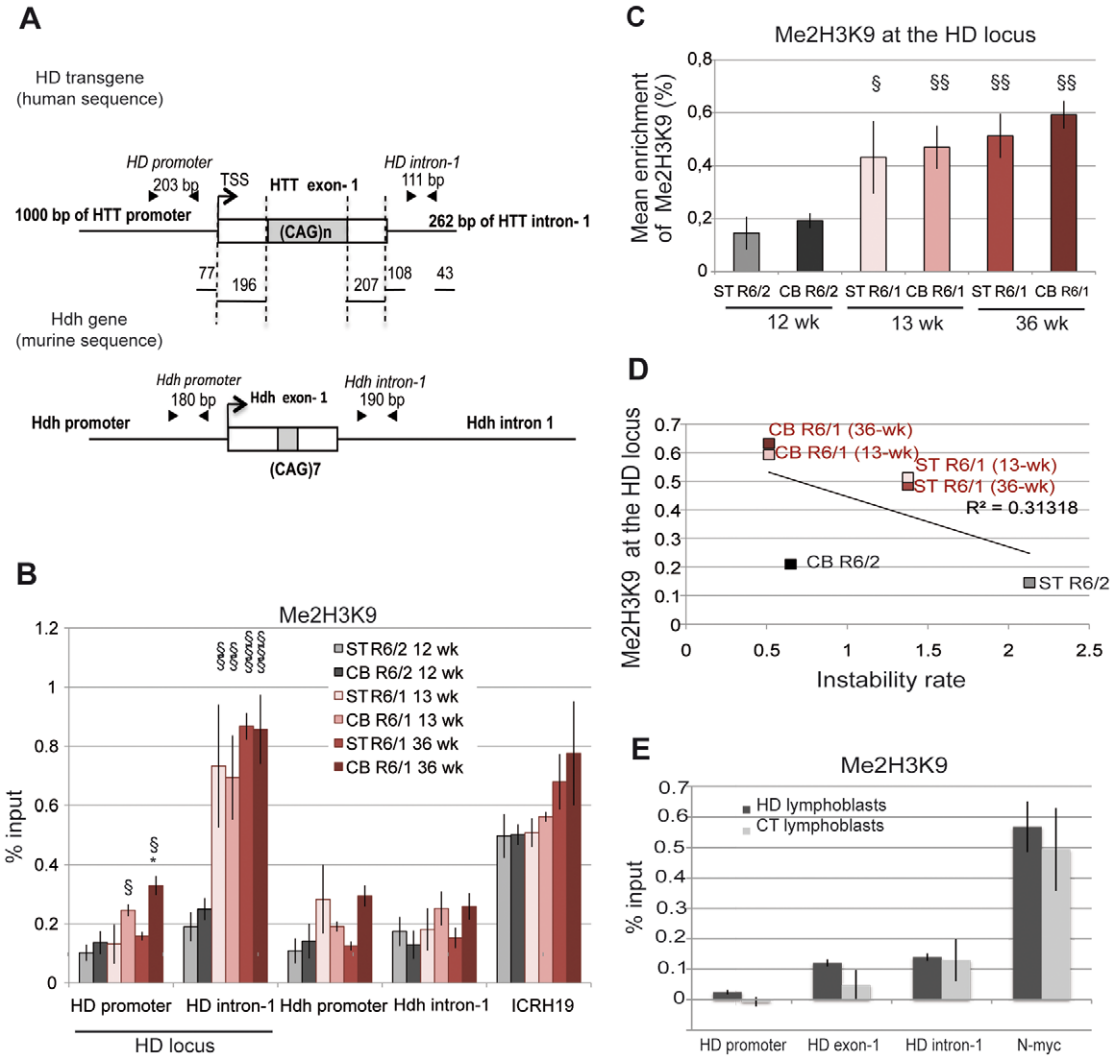
Me2H3K9 at the HD locus in R6/1 and R6/2 striatum and cerebellum. A. Schematic representation of the human HD transgene expressed in R6 mice and of the corresponding region in *Hdh*. The arrows show locations of the primers used to assess chromatin structure. B. ChIP analysis from the striatum (ST) and the cerebellum (CB) of R6/1 and R6/2 mice using antibody to Me2H3K9. R6/1 mice at early- and/or late-pathological stages (13- and 36-wk) and R6/2 mice at late-pathological stage (12-wk) were used. Error bars, sem; *, inter-tissue comparisons; §, inter-mouse line comparisons. *, p<0.05; §, p<0.05; §§, p<0.01, §§§, p<0.001. C. Global analysis of the level of Me2H3K9 at the HD locus. The mean levels of Me2H3K9 at the HD locus, corresponding to mean ChIP values at the HD promoter and HD intron-1, were calculated for the striatum and cerebellum of R6/1 mice at early and late-pathological stages (13- and 36-wk) and for the striatum and cerebellum of R6/2 mice at late pathological stage (12-wk). Error bars, sem; §, p<0.05; §§, p<0.01. D. Correlative analysis of CAG instability rates with mean levels of Me2H3K9 at the HD locus. R6/1 mice of 13 and 36 weeks and R6/2 mice of 12 weeks were used for correlation analysis E. ChIP analyses of the HD locus from lymphoblast cell lines derived from HD patients and control individuals (CT), using antibody to Me2H3K9. The values correspond to mean values calculated from ChIP performed from 4 HD cell lines and 2 control cell lines.

We investigated the hypothesis that the chromatin structure at the HD locus might be different in R6/1 and R6/2 tissues, which might underlie the different CAG instability rates. To explore this idea, we performed chromatin immunoprecipitation-PCR (ChIP-PCR) experiments and quantitatively assessed the level of histone marks associated with heterochromatin, euchromatin or transcriptionally active chromatin around the CAG expansion, as previously done for other TNRs associated to diseases [31], [37]–[39]. More specifically, we analyzed the striatum and the cerebellum of R6/1 and R6/2 mice. Tissue samples were sonicated to <500 bp to increase resolution of ChIP experiments (Figure S1). Primer pairs around the CAG tract, located ≈300 bp upstream of the repeats, within *HTT* proximal promoter (HD promoter), and ≈300 bp downstream of the repeats (HD intron-1), were selected to analyze the HD locus (Figure 2A). As controls, we included primers targeting regions expected to be euchromatic/transcriptionally active (the endogenous murine huntingtin gene *Hdh*, Hdh promoter and Hdh intron-1, Figure 2A) and heterochromatic/transcriptionally inactive (the *ICRH19* locus). We used R6/2 mice carrying 160 CAGs at late-pathological stage (12 week-old) and R6/1 mice carrying 130 CAGs at both early- and late-pathological stages (13 and 36 week-old, respectively).

**Figure 3:**
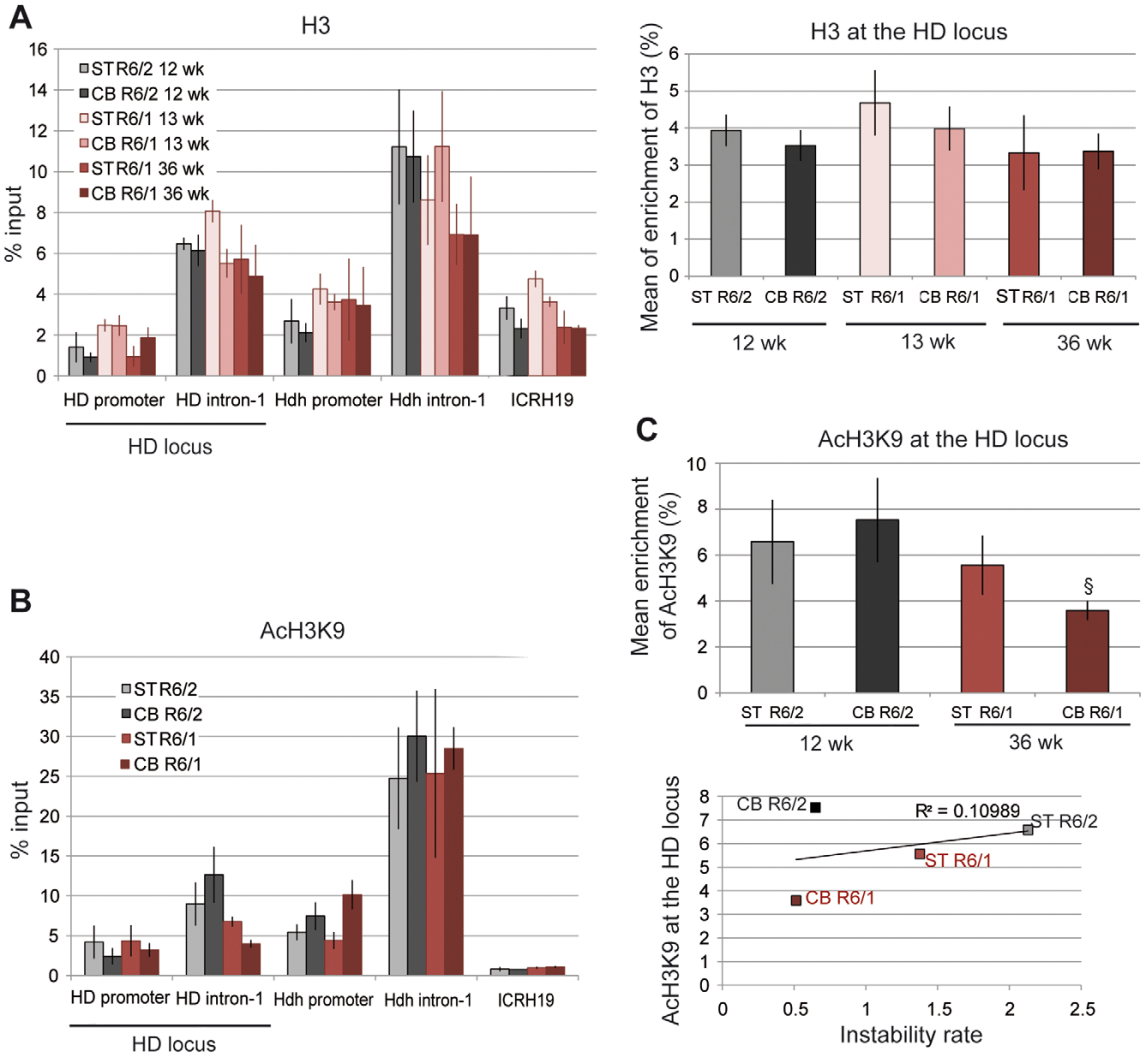
H3 and AcH3K9 at the HD locus in R6/1 and R6/2 striatum and cerebellum. A. Left panel. ChIP analyses from the striatum and the cerebellum of R6/1 and R6/2 mice using antibodies to H3. R6/1 mice at early and late-pathological stages (13- and 36-wk), and R6/2 mice at late pathological stage (12-wk) were used. Right panel. Global analysis of the level of H3 at the HD locus, assessed as in Figure 2. Error bars, sem. B. ChIP analyses from the striatum and the cerebellum of R6/1 mice at late-pathological stage (36-wk) and R6/2 mice at late-pathological stage (12-wk), using antibody to AcH3K9. C. Upper panel. Global analysis of the level of AcH3K9 at the HD locus, assessed as in Figure 2. Error bars, sem; §, p<0.05. Lower panel. Correlative analysis of CAG instability rates with mean levels of AcH3K9 at the HD locus. R6/1 mice of 36 weeks and R6/2 mice of 12 weeks were used for correlation analysis.

We first assessed the level of H3K9 dimethylation (Me2H3K9), a hallmark of heterochromatin. Me2H3K9 was 3- to 4-fold more elevated at HD intron-1 in R6/1 striatum and cerebellum when compared to R6/2 tissue-matched samples, regardless of age, and reached levels comparable to those measured at the *ICRH19* locus (Figure 2B). Me2H3K9 at the HD promoter was also increased in R6/1 cerebella with respect to R6/2 cerebella, though it was similar in striata (Figure 2B). Globally, Me2H3K9 level at the HD locus, as assessed by calculating the mean level at the HD promoter and HD intron-1, was significantly increased in R6/1 tissues as compared to R6/2 tissues (Figure 2C). The increased level was not dependent upon age. Moreover, the higher level of Me2H3K9 did not result from increased nucleosome occupancy, as measured by the level of total H3 at the HD locus, which was similar in R6/1 and R6/2 tissues (Figure 3A). Noticeably, unmodified H3 was lower at the HD promoter when compared to HD intron-1, consistent with reduced nucleosome occupancy at the region upstream of the transcription start site (TSS), a feature shared by many promoters [40]. We also assessed Me2H3K9 at the HD locus and at a control heterochromatic region (the N-myc locus) in HD and control human lymphoblasts. The level of Me2H3K9 at the HD locus was low as compared to the level at the N-myc locus, and similar in HD and control cells (Figure 2E). In addition, it was comparable to the level measured using R6/2 tissues. Together, these results suggest that the 3′ end of the HD transgene is heterochromatinized in R6/1 mice, due to position effect imposed on the transgene.

We then examined the correlation between somatic CAG instability and Me2H3K9 levels at the HD locus (Figure 2D). The R^2^ value was only 0.31. This is because Me2H3K9 level was similar in striatum and cerebellum, despite the fact that it was increased in R6/1 tissues relative to R6/2 tissues. Thus, Me2H3K9 level at the HD locus inversely correlates with mouse-line dependent instability, but not with tissue-selective instability.

### Chromatin is in a more transcriptionally active state at the HD locus in R6/2 tissues compared to R6/1 tissues

To further explore the chromatin structure at the HD locus of R6/1 and R6/2 mice, we assessed the level of the euchromatic mark acetyl H3K9 (AcH3K9) (Figure 3B). AcH3K9 was lowest at the *ICRH19* locus, which is consistent with heterochromatin. AcH3K9 at HD intron-1 was lower in R6/1 cerebellum with respect to R6/2 cerebellum but not significantly different in R6/1 and R6/2 striatum. AcH3K9 was consistently low at the HD promoter, likely resulting from reduced H3 levels (Figure 3A). No significant difference between tissues and mouse lines was seen for AcH3K9 at the HD promoter (Figure 3B). Globally, AcH3K9 at the HD locus was similar in the different tissues and mice, though it was slightly decreased in R6/1 cerebellum as compared to R6/2 cerebellum, and did not correlate with CAG instability rates (R^2^ = 0.11, Figure 3C).

**Figure 4:**
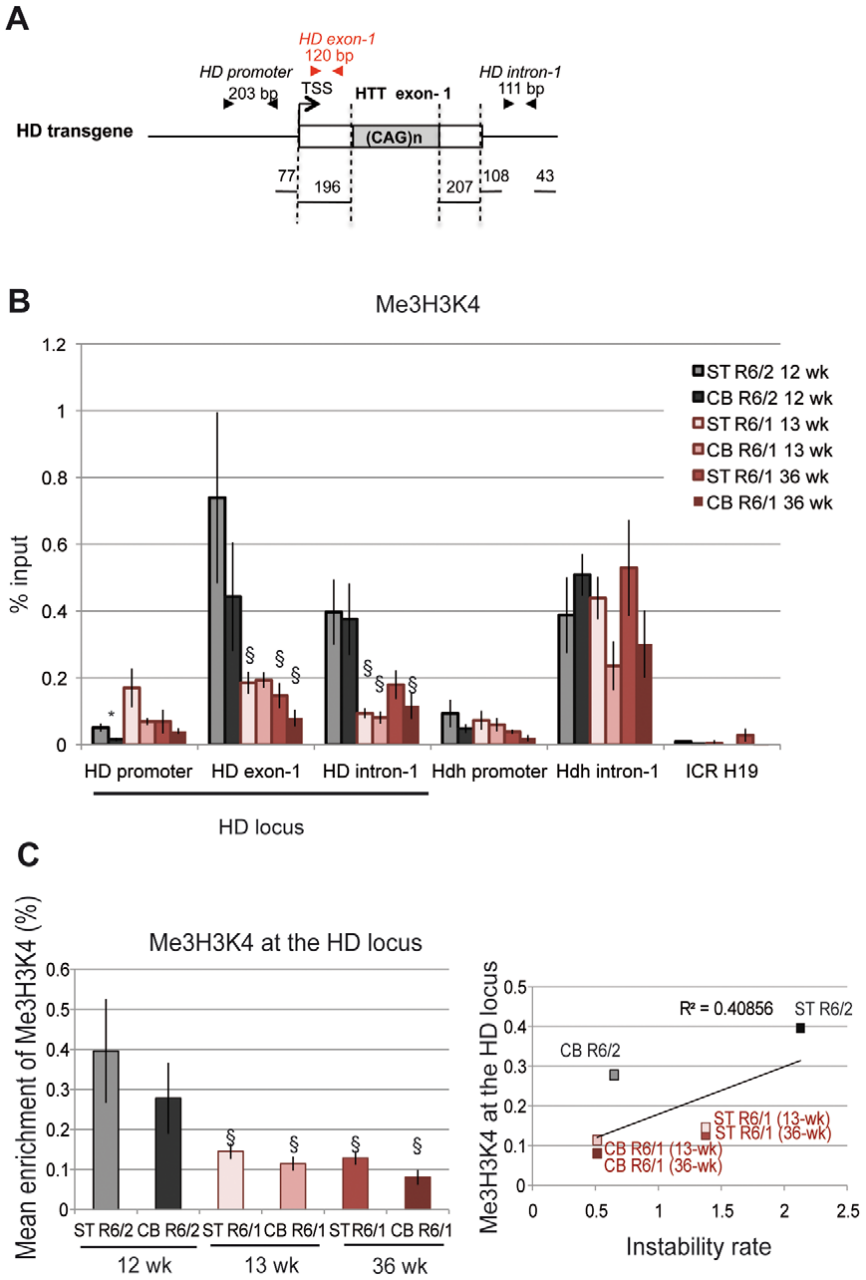
Me3H3K4 at the HD locus in R6/1 and R6/2 striatum and cerebellum. A. Schematic representation of the HD transgene expressed in R6 mice, showing location of primers used to amplify the region just downstream of the TSS in HD exon-1. B. ChIP analyses from the striatum and the cerebellum of R6/1 mice at 13- and 36-wk and from the striatum and cerebellum of R6/2 mice at 12-wk, using antibody to Me3H3K4. Error bars, sem; *, inter-tissue comparisons; §, inter-mouse line comparisons. *, p<0.05; §, p<0.05. C. Left panel. Global analysis of the level of Me3H3K4 at the HD locus. Me3H3K4 levels at the HD locus correspond to mean ChIP values at the HD promoter, HD exon-1 and HD intron-1. Error bars, sem; §, p<0.05. Right panel. Correlative analysis of CAG instability rates and Me3H3K4 levels at the HD locus. R6/1 mice of 13 and 36 weeks and R6/2 mice of 12 weeks were used for correlation analysis.

The euchromatic state is also characterized by histone post-translational modifications associated with transcription, including Me3H3K4, which is enriched at the TSS of active genes [41]. We assessed by ChIP the level of Me3H3K4 at the HD locus in the striatum and cerebellum of R6/1 and R6/2 mice (Figure 4). R6/1 mice were analyzed at 13 and 36 weeks of age and R6/2 mice at 12 weeks. To closely target the *HTT* TSS region, we included an amplicon corresponding to a fragment located between the TSS and CAG repeats (HD exon-1, Figure 4A). Consistent with the idea that nucleosome occupancy is low upstream of the TSS, Me3H3K4 was low at the HD promoter (Figure 4B). In contrast, Me3H3K4 at HD exon-1 and HD intron-1 was elevated, particularly in R6/2 tissues when compared to R6/1 tissues. Globally, Me3H3K4 at the HD locus was significantly increased in R6/2 tissues relative to R6/1 matched-tissues (Figure 4C), and this was not dependent upon mouse age. These results support the view that the chromatin at the HD locus is in a more transcriptionally active state in R6/2 than in R6/1 mice, and suggest that the transcriptional activity of the HD transgene is increased in R6/2 mice when compared to R6/1 mice. This is consistent with the results of Figure 2 suggesting that the chromatin at the HD locus is more heterochromatinized in R6/1 mice than in R6/2 mice. Thus, high Me3H3K4 and low Me2H3K9 levels at the HD locus correlate with high CAG instability levels of R6/2 mice. However, the correlation the R^2^ value between Me3H3K4 levels at the HD locus and CAG instability rates in striatum and cerebellum of R6/1 and R6/2 mice was only 0.40, due to comparable Me3H3K4 levels in mouse striatum and cerebellum (Figure 4C). Our results suggest that Me3H3K4 level does not correlate with tissue-selective instability, though it correlates with mouse line-dependent instability.

### Transcriptional activity of the HD transgene is increased in R6/2 mice compared to R6/1 mice

**Figure 5:**
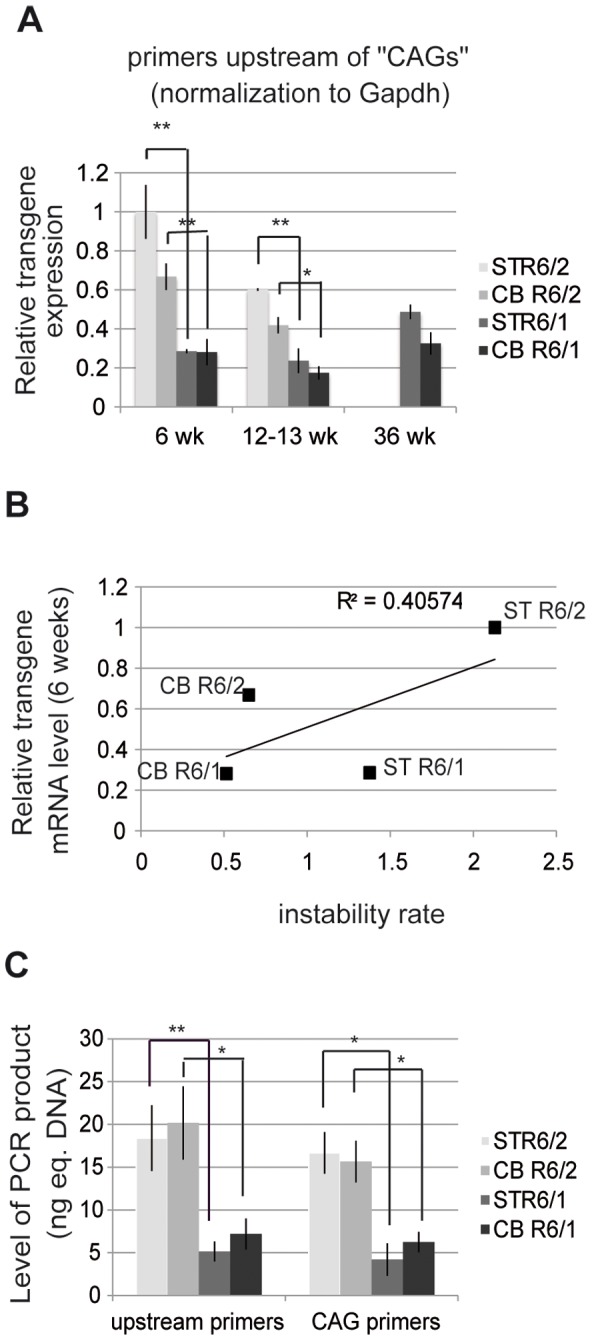
HD transgene expression in R6/1 and R6/2 striatum and cerebellum. A. Expression of the HD transgene in the striatum and in the cerebellum of R6/1 and R6/2 mice of various ages, as assessed by qRT-PCR using primers located upstream of CAG repeats in HD exon-1. Expression values were normalized to *Gapdh* expression. Error bars, sem; *, p<0.05; **, p<0.01. B. Correlative analysis of CAG instability rates and HD transgene mRNA levels at 6 weeks of age. C. Absolute quantification of HD transgene transcript levels, as assessed by qRT-PCR using primers upstream of the repeats (upstream primers) and primers amplifying the repeats (CAG primers), from the striatum and cerebellum of R6/1 and R6/2 mice of 6 weeks of age. Corresponding genomic DNAs were used as standards. Levels of PCR products are expressed as equivalent DNA (1 ng eq. DNA corresponds to the PCR signal obtained using 1 ng of DNA). Error bars, sem; *, p<0.05; **, p<0.01.

The above results suggest that HD transgene expression might be increased in R6/2 mice when compared to R6/1 mice. We measured the levels of HD transgene mRNA in R6 tissues by using quantitative RT-PCR (qRT-PCR) (Figure 5). The striatum and cerebellum of R6/1 mice and R6/2 mice were analyzed at both early- and end-pathological stages (*i.e.* 6, 13 and 36 weeks and 6 and 12 weeks, respectively). We selected primers targeting a region upstream of CAG repeats in HD exon-1 (Figure S2). Expression values were normalized relative to expression of *Gapdh* or *18S* expression (Figure 5A and Figure S3), which led to comparable results. At 6 weeks, transgene expression was 3- to 5-fold higher in R6/2 tissues in comparison to R6/1 tissue-matched samples, in accordance with the results above showing that chromatin structure at the HD locus is transcriptionally more active in R6/2 mice than in R6/1 mice. The differential transgene expression between R6/1 and R6/2 tissues was reduced over time, since HD transcript levels showed a tendency for decrease in R6/2 tissues. Transgene expression was not significantly different between the striatum and the cerebellum of either R6/1 or R6/2 mice. As a result, the correlation value R^2^ between HD transgene mRNA levels and CAG instability rates was only 0.40 (Figure 5B).

We further assessed transgene expression in striatum and cerebellum of 6 week-old R6/1 and R6/2 mice using an absolute quantification method with the corresponding genomic DNA as standards. This allowed for comparison between PCR product levels amplified with the above primers (located upstream of the repeats) and with primers encompassing the CAG repeats (“CAG” primers) (Figure 5C and S4). The level of transgene transcripts were comparable using both sets of primers, and increased in R6/2 tissues when compared to R6/1 tissue-matched samples using both sets of primers.

### RNA polymerase II level at the HD locus correlates with somatic CAG instability

**Figure 6:**
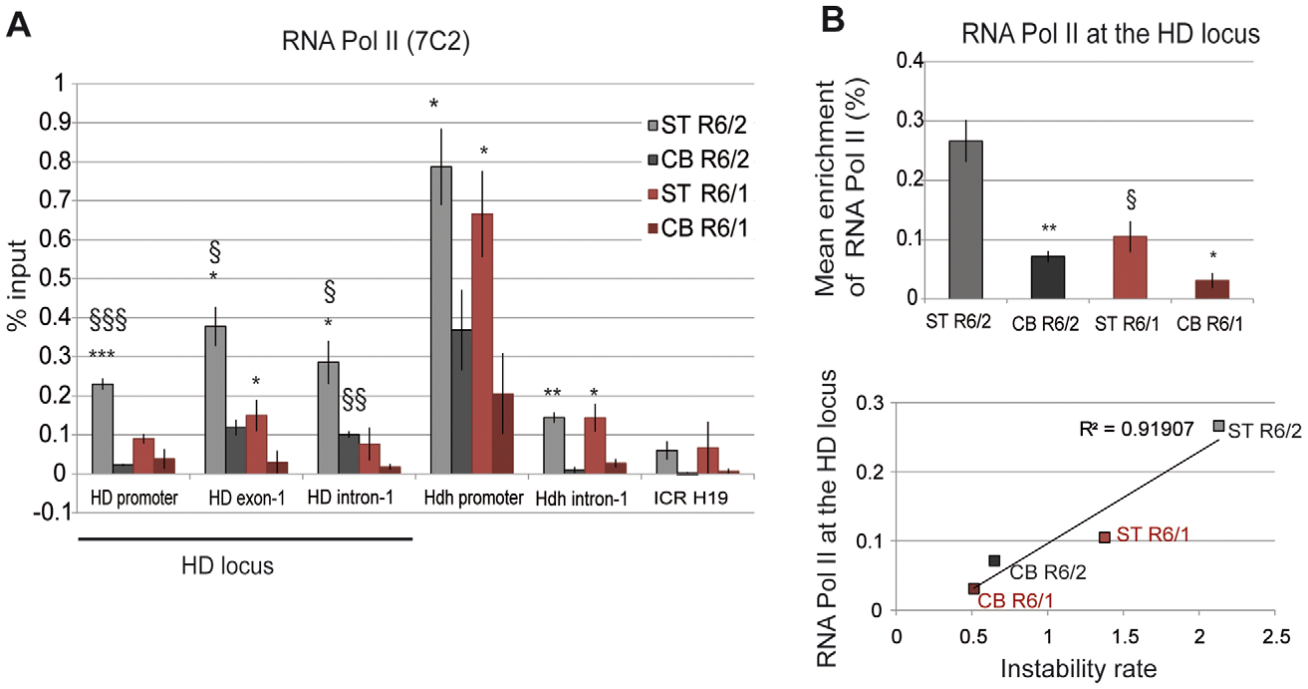
RNA Pol II at the HD locus in R6/1 and R6/2 striatum and cerebellum. A. ChIP analyses from the striatum and the cerebellum of R6/1 and R6/2 mice at late-pathological stages (36-wk and 12-wk, respectively) using an antibody to RNA Pol II (7C2), which recognizes phosphorylated and unphosphorylated forms of the enzyme. Error bars, sem; *, inter-tissue comparisons; §, inter-mouse line comparisons. *, p<0.05; §, p<0.05. B. Upper panel. Global analysis of the level of Me3H3K4 at the HD locus, assessed as in Figure 4. Error bars, sem; *, p<0.05; **, p<0.01 (inter-tissue comparison); §, p<0.05 (inter-mouse comparison). Lower panel. Correlative analysis of CAG instability rates and RNA Pol II levels at the HD locus. R6/1 mice of 36 weeks and R6/2 mice of 12 weeks were used for correlation analysis.

To investigate the relationship between transcription and somatic CAG instability, we performed ChIP from the striatum and cerebellum of R6/1 and R6/2 mice at late-pathological stages with the RNA Pol II 7C2 antibody, which recognizes phosphorylated and non phosphorylated forms of the polymerase [42]. RNA Pol II was distributed along the HD locus, peaking downstream of the TSS, at HD exon-1 (Figure 6A). Interestingly, RNA Pol II at the HD locus was higher in the striatum than in the cerebellum of both R6/1 and R6/2 mice, suggesting that the level of RNA Pol II at the HD region is regulated in a tissue-specific manner. Moreover, as previously found with Me2H3K9 and Me3H3K4 marks, RNA Pol II at the HD locus was increased (2- to 3-fold) in R6/2 tissues when compared to R6/1 tissue-matched samples (Figures 6A and 6B). As a result, RNA Pol II at the HD locus was highly correlated with CAG instability in R6 mice (R^2^ = 0.91, Figure 6B). RNA Pol II at control regions, including at *Hdh* regions, was also more abundant in the striatum than in the cerebellum of R6/1 and R6/2 mice (Figure 6A), further indicating that increased RNA Pol II at genes in striatum relative to cerebellum is tissue-specific. Together, these results suggest that a high level of RNA Pol II at the HD locus might contribute to CAG instability *in vivo*.

### Elongating RNA polymerase at the HD locus correlates with somatic CAG instability

**Figure 7:**
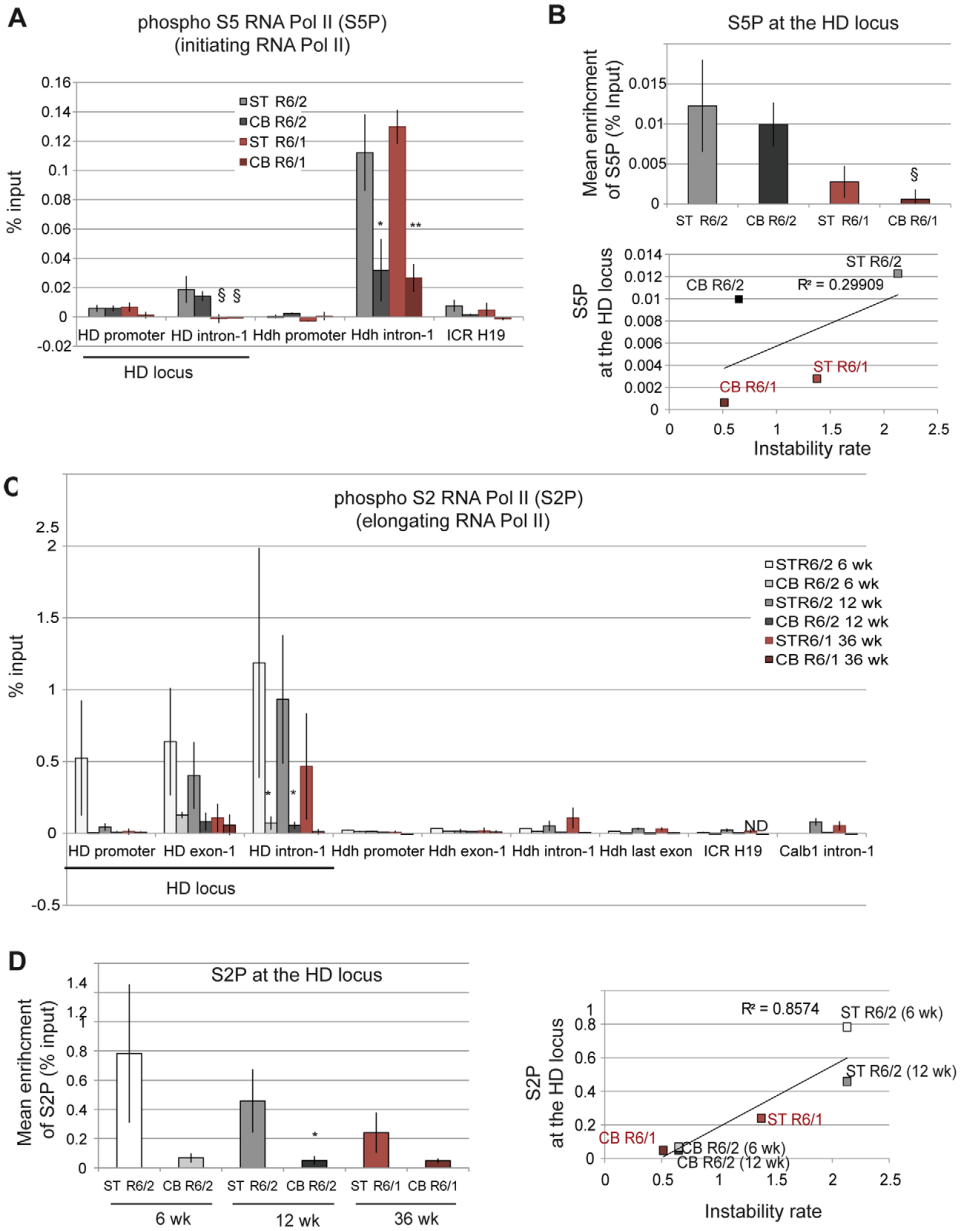
Phosphorylated RNA Pol II at the HD locus in R6/1 and R6/2 striatum and cerebellum. A. ChIP analyses from the striatum and the cerebellum of R6/1 and R6/2 mice using antibody to phosphorylated Ser5 RNA Pol II (S5P). R6/1 and R6/2 mice at late-pathological stages were used (36-wk and 12-wk, respectively). Error bars, sem; *, inter-tissue comparisons; §, inter-mouse line comparisons. §, p<0.05*; p<0.05; **, p<0.01. B. Upper panel. Global analysis of the level of S5P at the HD locus. Lower panel. Correlative analyses of CAG instability rates and S5P levels at the HD locus. R6/1 mice of 36 weeks and R6/2 mice of 12 weeks were used for correlation analysis. C. ChIP analyses from the striatum and the cerebellum of R6/1 and R6/2 mice using antibody to phosphorylated Ser2 RNA Pol II (S2P). R6/1 mice at late pathological stage (36-wk) and R6/2 mice at early- and late-pathological stages (6- and 12-wk) were used. *, p<0.05 (inter-tissue comparisons). D. Left panel. Global analysis of the level of S2P at the HD locus. Error bars, sem; §, p<0.05 (inter-mouse comparisons). Right panel. Correlative analyses of CAG instability rates and S2P levels at the HD locus. R6/1 mice of 36 weeks and R6/2 mice of 6 and 12 weeks were used for correlation analysis.

The activity of RNA Pol II during transcription is regulated by phosphorylation [43]–[45]. Ser5 phosphorylation of RNA Pol II is involved in transcription initiation, while Ser2 phosphorylation promotes transcription elongation. To discriminate between initiating and elongating RNA Pol II at the HD locus, we performed ChIP with antibodies to phosphorylated Ser5 RNA Pol II and phosphorylated Ser2 RNA Pol II (Figure 7A). As for Me3H3K4, which is associated with transcription initiation, the level of initiating RNA Pol II at the HD locus was increased downstream of the TSS, was comparable in the striatum and cerebellum of HD mice and was globally increased in R6/2 mice when compared to R6/1 mice (Figure 7A and 7B). As a result, initiating RNA Pol II was poorly correlated with somatic CAG instability in R6 mice (R^2^ = 0.30; Figure 7B). Elongating RNA Pol II at the HD locus peaked downstream of the TSS, particularly at the HD intron-1, but was more elevated in striata than in cerebella of both R6/1 and R6/2 mice (Figure 7C). In addition, the increase was more pronounced in R6/2 striatum relative to R6/1 striatum (Figures 7C and 7D). As a result, CAG instability rates were highly correlated with the level of elongating RNA Pol II at the HD locus (R^2^ = 0.86; Figure 7D). These results suggest that the dynamics of transition from initiation to elongation is tissue-specific and might contribute to tissue-selective CAG instability. Elongating RNA Pol II at the HD locus appeared abnormally high because we did not detect such levels at similar regions of control active genes, including *Hdh* (Figure 7C and S5). This might suggest that CAG repeats impair the dynamics of transcription. Together, these results suggest that high elongating RNA Pol II levels, resulting from tissue-specific regulation of elongation dynamics, and high transcriptional activity at the HD locus, as seen in R6/2 striatum, increase the propensity for repeat instability.

**Figure 8:**
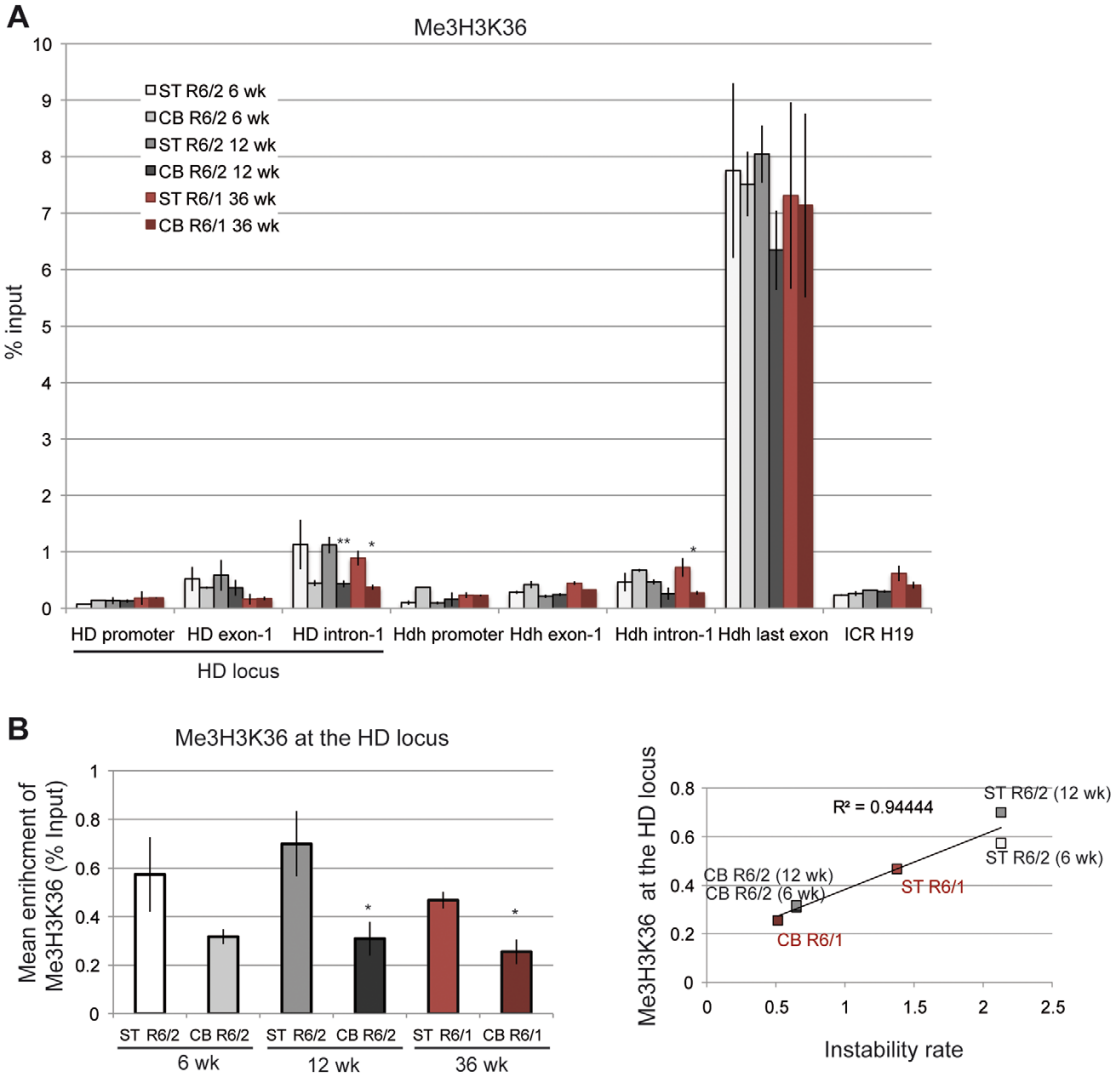
Me3H3K36 at the HD locus in R6/1 and R6/2 striatum and cerebellum. A. ChIP analyses from the striatum and the cerebellum of R6/1 and R6/2 mice using antibody to Me3H3K36. R6/1 mice at late pathological stage (36-wk) and R6/2 mice at early- and late-pathological stages (6- and 12-wk) were used. Error bars, sem; *, p<0.05; **, p<0.01 (inter-tissue comparisons). B. Left panel. Global analysis of the level of S2P at the HD locus. Error bars, sem; *, p<0.05 (inter-tissue comparisons). Right panel. Correlative analyses of CAG instability rates and S2P levels at the HD locus. R6/1 mice of 36 weeks and R6/2 mice of 6 and 12 weeks were used for correlation analysis.

We further examined the correlation between transcription elongation and somatic CAG instability by performing ChIP with an antibody to Me3H3K36, a histone mark associated with transcription elongation and termination [41], [46]. Me3H3K36 is particularly elevated at the 3′ ends of transcribed genes, and, accordingly, was elevated at the last *Hdh* exon (Figure 8A). Me3H3K36 at HD intron-1 was lower than at *Hdh* last exon, but interestingly was significantly increased in striatal tissues in both R6/1 and R6/2 mice when compared to corresponding cerebellar tissues. Globally, Me3H3K36 level at the HD locus was increased in striatum when compared to cerebellum (Figure 8B), and strongly correlated with somatic CAG instability in HD mice (R^2^ = 0.94; Figure 8B). Since the tissue selectivity of somatic CAG instability is conserved between R6 mice and HD patients, these results, taken together, suggest that transcription elongation regulation might contribute to HD somatic CAG instability *in vivo*.

## Discussion

*In vitro* or cell-based studies support a role for transcription in CAG/CTG instability [20]–[24]. However, the expression level of TNR genes does not correlate with tissue-selective somatic instability *in vivo* [26], [27], [47], [48]. Thus, the role of transcription in somatic CAG instability has remained unclear (reviewed in [1]). Our results show that despite HD transgene expression being similar between striatum and cerebellum from both R6/1 and R6/2 mouse lines, these tissues present very different levels of CAG instability (Figure 5). In addition, the level of histone modifications associated with heterochromatin (Me2H3K9), euchromatin (AcH3K9) and transcription initiation (Me3H3K4) at the HD locus was comparable in the striatum and cerebellum of HD mice (Figure 2, Figure 3 and Figure 4). Remarkably however, the level of a histone mark and RNA Pol II form associated with transcription elongation (*e.g.* Me3H3K36 and phosphorylated Ser2 RNA Pol II, respectively) correlated strongly with CAG instability in tissues of R6/1 and R6/2 mice (Figure 7 and Figure 8). Thus, our data suggest a specific role for transcription elongation regulation in modulating tissue-selective CAG/CTG instability in HD. Moreover, comparison between mouse lines shows that increased CAG instability in R6/2 tissues relative to R6/1 matched-tissues correlates with increased chromatin accessibility and transcription of the HD transgene (Figure 2, Figure 4 and 5). This suggests that integration site of the transgene influences repeat instability, affecting chromatin structure and transcriptional activity.

Previous studies have shown that TNRs can induce a heterochromatinization process, consisting in formation and spreading of heterochromatin at TNR loci. This process has been described in the case of DM1, FRDA and FXS [29], [31], [37], [38], [49]–[51]. Me2H3K9 was not significantly increased at the HD locus in lymphoblasts derived from HD patients, suggesting absence or limited heterochromatinization of the locus (Figure 2). CAG expansion implicated in HD rarely exceeds 80 repeats, whereas TNRs associated with DM1, FRDA and FXS are typically larger (often reaching several hundred to thousand units), which could contribute to their high propensity to induce heterochromatinization, and to transcriptional silencing in the case of FRDA and FXS [29], [51]–[53]. Consistent with transcriptional repression, the levels of histone marks and RNA Pol II associated with transcription initiation and elongation are decreased at the FRDA locus in FRDA cells [37], [38], [54]. However, the effect of heterochromatinization on TNR instability has remained unclear. Our results suggest that heterochromatinization of the HD locus, a situation mimicked in R6/1 tissues, correlates with decreased somatic CAG instability. Whether heterochromatinization would limit TNR instability at the expense of transcription in FRDA or FXS is an intriguing possibility. If true, reversal of heterochromatinization through histone deacetylase (HDAC) inhibition, a process currently considered to alleviate GAA-repeat induced gene silencing in FRDA [55], might in turn increase repeat instability. Surprisingly, depletion of HDACs reduced TNR expansion in yeast and a plasmid-based cell model [56]. However, the effect was likely mediated through alteration of non-histone enzyme activities, including the Sae2 nuclease.

Factors involved in various DNA repair mechanisms, including MMR, BER and NER, contribute to CAG/CTG instability *in vivo*, most likely through aberrant processing of stable secondary structures formed at repeats, such as hairpins or slip-outs [1], [14], [16], [17], [19], [57]–[60]. Accessibility of DNA to repair factors increases when chromatin is in an open conformation. We therefore tested the hypothesis that CAG/CTG instability and chromatin accessibility might be correlated. Although Me2H3K9 at the HD locus was increased in R6/2 tissues when compared to R6/1 tissues, it was similar in the striatum and in the cerebellum of R6 mice, indicating that chromatin accessibility is unlikely to underlie tissue-selective HD somatic CAG instability (Figure 2). Instead, our data suggest a role for transcription, and more specifically for transcription elongation, in somatic CAG instability in HD. Events associated with transcription initiation were only partially correlated with CAG instability rates in R6 mouse tissues, since Me3H3K4 and phosphorylated Ser5 RNA Pol II levels at the HD locus were increased in R6/2 when compared to R6/1 tissues, but were comparable in striatum and cerebellum (Figure 4 and Figure 7). In contrast, events associated with transcription elongation were strongly correlated with somatic CAG instability. Specifically, Me3H3K36 and phosphorylated Ser2 RNA Pol II levels at the HD locus were increased in striatum relative to cerebellum as well as in R6/2 tissues as compared to R6/1 tissues (Figure 7 and Figure 8). Interestingly, a previous study based on a cell model allowing for detection of contraction events at CAG/CTG repeats showed that the elongation factor TFIIS contributes to repeat instability [15]. In addition, inhibition of the proteasome and downregulation of BRCA1/BARD1 proteins, which modulate RNA Pol II activity and transcription elongation, decreased CAG/CTG instability in the treated cells [15]. It remains to be determined whether these proteins as well as other factors controlling the dynamics of transcription elongation, including positive transcription-elongation factor-b (P-TEFb), negative elongating factor (NELF) or DRB sensitivity-inducing factor (DSIF), a factor composed of SPT4 and SPT5 [61]–[63], contribute to tissue-selective CAG instability in HD. Interestingly, a recent study showed that depletion of SPT4 in yeast and mammalian cells selectively prevents transcription of CAG-expanded genes, thereby reducing toxicity of the gene products [64]. Thus, targeting transcription elongation might reduce both repeat instability and toxicity.

Total RNA Pol II at the HD locus and at the promoter-proximal region of the murine *Hdh* gene were increased in the striatum when compared to the cerebellum (Figures 5). This suggests that transcriptional activity at the Huntingtin gene is differently regulated in the striatum and in the cerebellum in both mice and humans. However, and in accordance with previous studies showing that normal and mutant Huntingtin expression is widespread in many tissues and comparable in striatum and cerebellum, HD transgene expression was not different in the striatum and cerebellum, irrespective of mouse age or line (Figure 5) [26], [27], [47], [48], [65]. *HTT* mRNA processing might be regulated in a tissue-specific manner, as previously suggested [66], [67]. As a result, despite increased transcriptional activity in the striatum as compared to the cerebellum, *HTT* mRNA levels could be similar in the two tissues, due to increased *HTT* mRNA stability in the cerebellum. Alternatively, the dynamics of *HTT* transcription might be different in striatum and cerebellum, which cannot be examined by measuring bulk mRNA levels. Increasing evidence shows that genes can be expressed through alternative modes, ranging from bursts of transcription to more continuous smooth mode [68], [69]. Whether transcriptional mode can be regulated in a tissue-specific manner is currently unknown and cannot be easily assessed. The differential levels of RNA Pol II at *HTT* promoter-proximal region might suggest that the regulation of the transition from initiation to productive elongation is different in striatum and cerebellum, supporting the view that the dynamics of *HTT* transcription is different in the two tissues [70]. Whether *HTT* is expressed through a bursting mode in the striatum -promoting CAG instability, and a more continuous mode in the cerebellum –limiting repeat instability, is an attractive hypothesis.

The dynamics of transcription across CAG expansion might be impaired, particularly in the striatum where elongating RNA Pol II at the HD locus is elevated (Figure 7). Using an *in vitro* approach, it was recently shown that G/C-rich sequences induce transcription stalling, due to formation of stable R-loops during elongation [71]. Cell-based studies showed that increased transcriptional activity at genes with TNRs or bidirectional transcription across CAG/CTG repeats promotes formation of R-loops, leading to increased TNR instability [22]–[24], [72]. How then would R-loops promote TNR instability is currently unknown, but it has been hypothesized that arrest of elongating RNA Pol II at non canonical DNA structures might result in gratuitous transcription-coupled nucleotide excision repair (TCR) [73]. Supporting this view, it was shown that proteins of the NER/TCR pathway, including CSB, XPG and XPA, reduce transcription-mediated instability of CAG/CTG repeats in a cell model allowing for detection of contraction events [15], [20]. Accordingly, somatic CAG instability was reduced in post-mitotic tissues, including brain tissues, in spinocerebellar ataxia type 1 mice deficient for *Xpa* [19]. The idea that the propensity for formation of stable R-loops is increased in somatic tissues presenting high CAG instability rates, such as the striatum, is an intriguing hypothesis.

In conclusion, our results indicate that increased chromatin accessibility and transcription provide a context favoring somatic CAG instability but do not underlie the tissue selectivity of the process. Our data suggest that transcription elongation specifically contributes to tissue-specific CAG instability. It is tempting to speculate that the dynamics of transcription elongation of the *HTT* gene is regulated in a tissue-specific manner, influencing the risk for CAG instability.

## Materials and Methods

### Mouse lines and breeding

Hemizygous R6/1 (130 CAG) and R6/2 (160 CAG) mice from the Jackson Laboratory were both maintained on a mixed CBAxC57BL/6 genetic background. The experiments were approved by the ethical committee C.R.E.M.E.A.S (Comité Régional d'Ethique en Matière d'Expérimentation Animale de Strasbourg).

### Cell lines

Lymphoblasts from control individuals (GM14907, GM06903) and HD patients (GM04868, GM04282, GM04724, GM04846) were obtained from the Coriell Cell Repository. The size of CAG repeats in HD cell lines is 49/13, 74/17, 68/18 and 50/48, respectively. Cell lines were grown in RPMI 1640 medium without Hepes supplemented with 15% fetal calf serum and gentamycin under standard conditions.

### CAG repeat sizing and analysis

CAG repeat size was determined as previously described [35], [36]. Briefly, PCR products containing the CAG repeats were analyzed using the ABI Prism 3100 DNA analyzer instrument and GeneScan and Genotyper softwares. The variation of repeat size corresponds to the amplitude of Genescan profile and was determined by calculating the number of peaks above 10% of the maximum fluorescent peak intensity. The striatum and cerebellum of the same mice were analyzed at each time point and 4 to 8 different mice at each time point were analyzed.

### ChIP

ChIP experiments were performed essentially as described [36]. Briefly, for each experiment, striata and cerebella from 3 to 5 transgenic mice were pooled, cut into small fragments, fixed in 1% formaldehyde and incubated for 10 min at room temperature. Cross-linking was stopped by the addition of glycine to final concentration 0.125 M. Tissue fragments were washed with cold PBS supplemented with protease inhibitors [36]. Phosphatase inhibitors were included when using antibody to phospho Ser2 or Ser5 RNA Pol II. The tissues were then homogenized in sonication buffer, and lysates were sonicated to obtain DNA fragments <500 bp and centrifuged, as described [36]. The soluble chromatin fraction was pretreated with protein A Agarose/Salmon Sperm DNA (Millipore) for 1 h at 4°C. Subsequently, samples were incubated overnight at 4°C with antibodies to AcH3K9 (ab10812, Abcam), Me2H3K9 (ab1220, Abcam), H3 (ab1791, Abcam), Me3H3K4 (ab1012, Abcam), Me3H3K36 (ab9050, Abcam), phospho S5 RNA Pol II (ab5131, Abcam), phospho S2 RNA Pol II (ab5095, Abcam) and 7C2 RNA Pol II antibody [42]. Protein A Agarose/Salmon Sperm DNA was then added and the mixture was incubated for 3 h at 4°C. Agarose beads were washed, protein-DNA complexes were eluted from the beads and decrosslinked, and DNA recovered by phenol chloroform extraction and ethanol precipitation after treatment with ribonuclease A (Abcam) and proteinase K, as described [36]. Typically, 2 to 3 antibodies were used per experiment, and immunoprecipitation with each antibody was duplicated. 2 to 4 independent ChIP experiments were performed with each antibody. The results are expressed as percentage of input, and correspond to mean values calculated from the different experiments. ChIP experiments from cell lines were performed using 5×10^6^ cells per ChIP and the procedure described above. A typical ChIP experiment was performed using 4 HD cell lines and 2 control cell lines. Immunoprecipitations with each cell line were performed with antibodies to Me2H3K9 (ab1220, Abcam) and RNA Pol II (7C2) in duplicates, and 2 independent ChIP experiments were performed. The results, expressed as percentage of input, correspond to a representative experiment and are calculated by averaging values from the 2 control cells and from the 4 HD cells. The PCR primers used are listed in Figure S2.

### RNA extraction and real-time RT–PCR analysis

Striata and cerebella were dissected, snap frozen and stored at −80°C. Total RNA was prepared from frozen tissues using the RNeasy Mini Kit (Qiagen). cDNA were prepared using 1 mg of total RNA, random hexamers and SuperScriptII reverse transcriptase (Invitrogen) according to the manufacturer instructions. Quantitative RT-PCRs were performed on a Light-Cycler instrument (Roche) to assess expression levels of HD transgene. The SYBR PCR master mix (Qiagen) was used to assess the level of HD transgene mRNA using the primers upstream of CAG repeats. Specific conditions were used to assess the level of HD transgene mRNA using primers amplifying the repeats, as previously described [36]. Briefly, the Herculase Hotstart DNA Polymerase (Stratagene) was used according to recommendation of manufacturer, and 8% DMSO and Sybr green (Molecular probe) were included in the reaction. The PCR reactions were performed on a Light Cycler instrument (Roche). DNA was inactivated for 3 min at 98°C, and 45 cycles consisting in 40 seconds at 98°C, 30 seconds at 60°C and 2 min at 72°C were performed. As a control, PCR reactions were also performed in the absence of reverse transcriptase. Data were normalized to expression of *Gapdh* or *18S* housekeeping genes. For absolute quantifications, standard curves were generated using genomic DNA and used to calculate amounts of PCR products. cDNA samples prepared from the striatum and cerebellum of R6/1 and R6/2 mice were analyzed using corresponding genomic DNA. All experiments were performed at least three times in biological triplicates. The PCR primers used are provided in Figure S2.

### Statistical analyses

ANOVA followed by Newman Keuls tests were used for statistical analyses.

## Supporting Information

Figure S1 Examples of sonicated DNA prepared from the striatum (ST) and the cerebellum (CB) of R6/1 and R6/2 mice.
(PDF)

Figure S2 Sequence of primers used for ChIP experiments and quantitative RT-PCR.
(PDF)

Figure S3 HD transgene expression in R6/1 and R6/2 striatum and cerebellum according to age. The same samples as shown in Figure 5A were analyzed, except that expression data were normalized to *18S*. Transgene expression was assessed using the primers upstream of CAG repeats.
(PDF)

Figure S4 A. Real time quantitative amplification of CAG expansion using DNA prepared from striatum and cerebellum of R6/1 and R6/2 mice of 6 weeks of age as standards. Upper panel. Profiles showing that the CAG primers amplify a specific product in a quantitative manner when using DNA from the striatum and cerebellum of R6/1 mice. 3.125 to 50 ng of genomic DNA was used for standard curves. Amplification of cDNA prepared from RNA of striatum and cerebellum of R6/1 and R6/2 mice falls within the standard curves. CT, RNA samples that were not treated with reverse transcriptase. Lower panel. Graph showing that the relative DNA concentration calculated by the Light Cycler software and using the CAG primers is proportional to the initial quantity of DNA, similar between striatum and cerebellum and similar between R6/1 and R6/2 mice. Thus, amplification of the same amount of genomic DNA of R6/1 and R6/2 mouse tissues results in similar levels of PCR products. B. Genomic DNA of R6/1 and R6/2 striatum and cerebellum was amplified using the primers upstream of the repeats. Amplification of the same amount of genomic DNA of R6/1 and R6/2 mouse tissues also results in similar levels of PCR products using the upstream primers.
(PDF)

Figure S5 Comparison of HD transgene expression and *Hdh* expression in the striatum and cerebellum of R6/1 and R6/2 mice of 6 weeks. Primers located upstream of the CAG repeats in exon-1 were used in both cases. An absolute quantification method was used to compare transgene and *Hdh* mRNA levels, using genomic DNA from the striatum and cerebellum of R6/1 and R6/2 mice. Levels of PCR products are expressed as equivalent DNA (1 ng eq. DNA corresponds to the PCR signal obtained using 1 ng of DNA). As expected, *Hdh* mRNA levels are similar in R6/1 and R6/2 matched-tissues, and similar in striatum and cerebellum.
(PDF)
