# Supplementary figures and images for: Transcription Elongation and Tissue-Specific Somatic CAG Instability
Source: PLoS Genet. 2012 Nov 29;8(11):e1003051. doi: 10.1371/journal.pgen.1003051 (PMC3510035; doi:10.1371/journal.pgen.1003051)

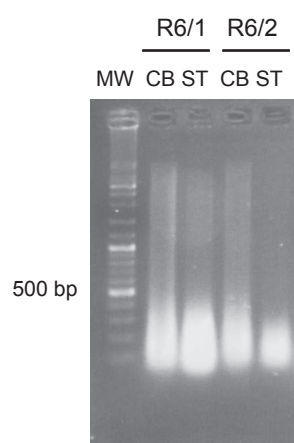

Fig S1

Supplement: Figure S1 — Examples of sonicated DNA prepared from the striatum (ST) and the cerebellum (CB) of R6/1 and R6/2 mice. (PDF) [file pgen.1003051.s001.pdf]

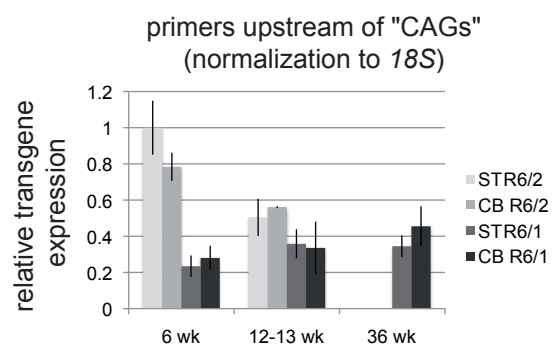

Fig.S3

Supplement: Figure S3 — HD transgene expression in R6/1 and R6/2 striatum and cerebellum according to age. The same samples as shown in Figure 5A were analyzed, except that expression data were normalized to 18S. Transgene expression was assessed using the primers upstream of CAG repeats. (PDF) [file pgen.1003051.s003.pdf]

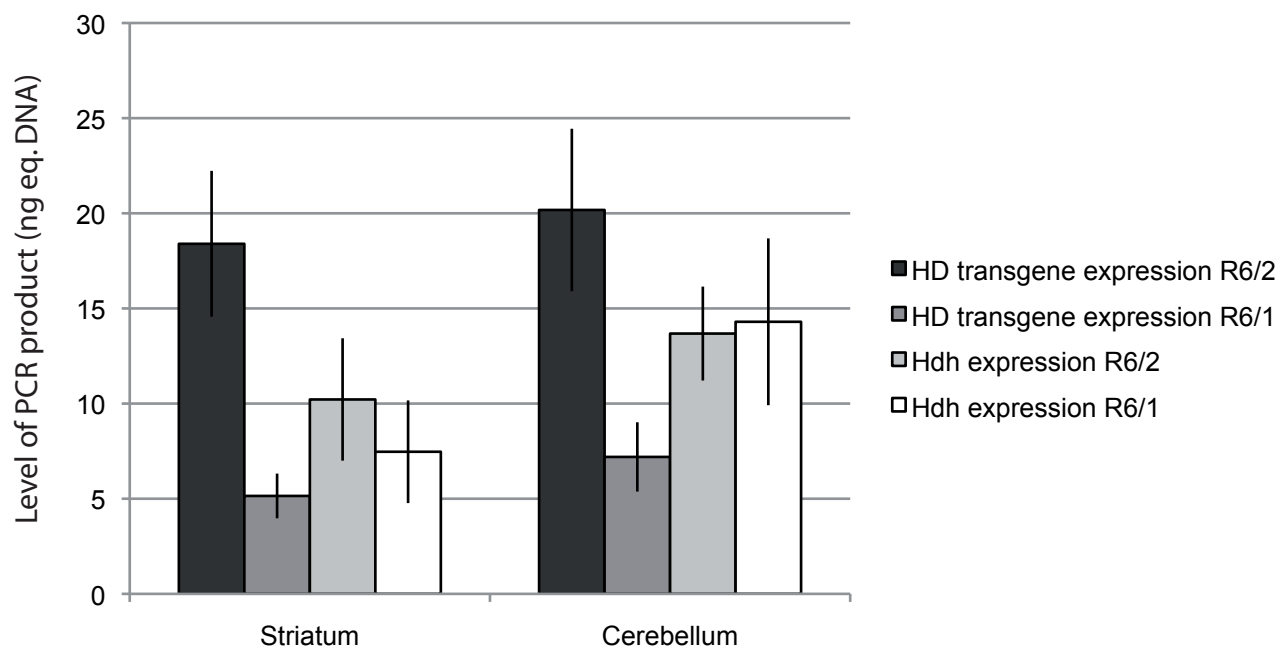

Fig.S5

Supplement: Figure S5 — Comparison of HD transgene expression and Hdh expression in the striatum and cerebellum of R6/1 and R6/2 mice of 6 weeks. Primers located upstream of the CAG repeats in exon-1 were used in both cases. An absolute quantification method was used to compare transgene and Hdh mRNA levels, using genomic DNA from the striatum and cerebellum of R6/1 and R6/2 mice. Levels of PCR products are expressed as equivalent DNA (1 ng eq. DNA corresponds to the PCR signal obtained using 1 ng of DNA). As expected, Hdh mRNA levels are similar in R6/1 and R6/2 matched-tissues, and similar in striatum and cerebellum. (PDF) [file pgen.1003051.s005.pdf]
