# Supplementary material for: Transcription Elongation and Tissue-Specific Somatic CAG Instability
Source: PLoS Genet. 2012 Nov 29;8(11):e1003051. doi: 10.1371/journal.pgen.1003051 (PMC3510035; doi:10.1371/journal.pgen.1003051)
Supplement: Figure S2 — Sequence of primers used for ChIP experiments and quantitative RT-PCR. (PDF) [file pgen.1003051.s002.pdf]

Sequence of primers for ChIP

| | Forward (5' to 3') | Reverse (5' to 3') |
|---|---|---|
| HD promoter (h) | ACACTTCACACACAGCTTCG | AGCATGATTGACAGCCCTAG |
| HD exon-1 (h) | GCTCAGGTTGTGCTTTTACC | TCATCAGCTTTTCCAGGGTC |
| HD intron-1 (h) | AGAGACAGAGTGACCCAGC | ACAAGGGAAGACCCAAGTGA |
| Hdh promoter (m) | CTTTTCTATCGCTGGTGCCA | GCTAGTTTAAGACTCTCCGC |
| Hdh exon-1 (m) | AGAGCCCCATTCATTGCCTT | AAACGACTTGAGCGACTCGA |
| Hdh intron-1 (m) | AACACTGTTCCCTGTCCAGA | CGTTCCCTAACTTCGCAAAC |
| Hdh last exon (m) | CTGGTTGCCACAGACTTCTA | AGCTGCCTTCTTTCAGCCTT |
| ICR H19* (m) | CCGAGAAAATAGCCATTGCCTACAGT | CATGTTCCTTTGAGTCCTGGGTGTA |
| Calb intron-1 (m) | TCTTCCCTTCGACTGTGCTT | GAAAGGTGAACGCTTACAGC |
| N-Myc** (h) | GGCTCTGTGAGGAGGCAAGGTG | GCTCTCTATTTGGAGTGGCGGG |

Sequence of primers for quantitative RT-PCR

| | Forward (5' to 3') | Reverse (5' to 3') |
|---|---|---|
| Upstream "CAGs" (h) | GCTCAGGTTGTGCTTTTACC | TCATCAGCTTTTCCAGGGTC |
| "CAGs"*** (h) | ATGAAGGCCTTCGAGTCCCTCAAGTCCTTC | GGCGGCTGAGGAAGCTGAGGA |
| HD intron-1 (h) | AGAGACAGAGTGACCCAGC | ACAAGGGAAGACCCAAGTGA |
| Gapdh (m) | TTGTGATGGGTGTGAACCAC | TTCAGCTCTGGGATGACCTT |
| 18S (m) | GCGAGTACTCAACACCAACA | CCTCAACACCACATGAGCAT |

h, human sequence; m, mouse sequence
* According to Ling et al. (Science, 2006)
** According to De Biase et al. (PLoS One, 2009)
*** According to Mangiarini at al. (Nat. Genet., 1997)

Fig. S2
