# Supplementary material for: Transcription Elongation and Tissue-Specific Somatic CAG Instability
Source: PLoS Genet. 2012 Nov 29;8(11):e1003051. doi: 10.1371/journal.pgen.1003051 (PMC3510035; doi:10.1371/journal.pgen.1003051)
Supplement: Figure S4 — A. Real time quantitative amplification of CAG expansion using DNA prepared from striatum and cerebellum of R6/1 and R6/2 mice of 6 weeks of age as standards. Upper panel. Profiles showing that the CAG primers amplify a specific product in a quantitative manner when using DNA from the striatum and cerebellum of R6/1 mice. 3.125 to 50 ng of genomic DNA was used for standard curves. Amplification of cDNA prepared from RNA of striatum and cerebellum of R6/1 and R6/2 mice falls within the standard curves. CT, RNA samples that were not treated with reverse transcriptase. Lower panel. Graph showing that the relative DNA concentration calculated by the Light Cycler software and using the CAG primers is proportional to the initial quantity of DNA, similar between striatum and cerebellum and similar between R6/1 and R6/2 mice. Thus, amplification of the same amount of genomic DNA of R6/1 and R6/2 mouse tissues results in similar levels of PCR products. B. Genomic DNA of R6/1 and R6/2 striatum and cerebellum was amplified using the primers upstream of the repeats. Amplification of the same amount of genomic DNA of R6/1 and R6/2 mouse tissues also results in similar levels of PCR products using the upstream primers. (PDF) [file pgen.1003051.s004.pdf]

A qRT-PCR of the amplicon containing the CAG expansion (using CAG primers)

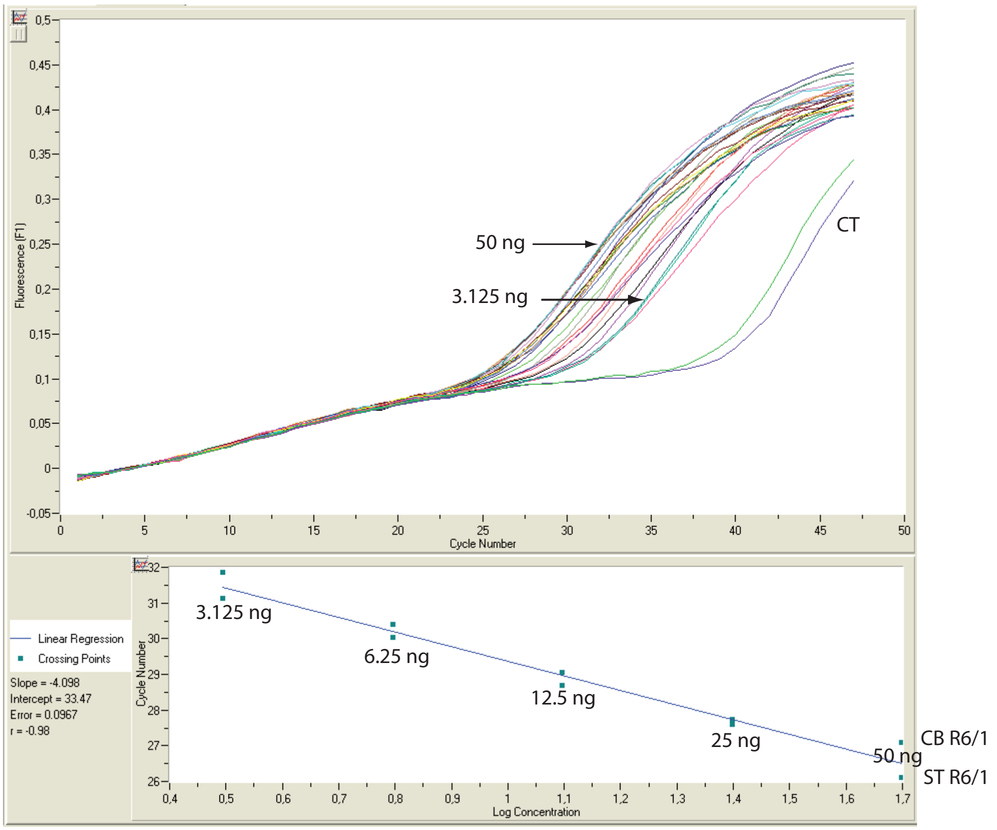

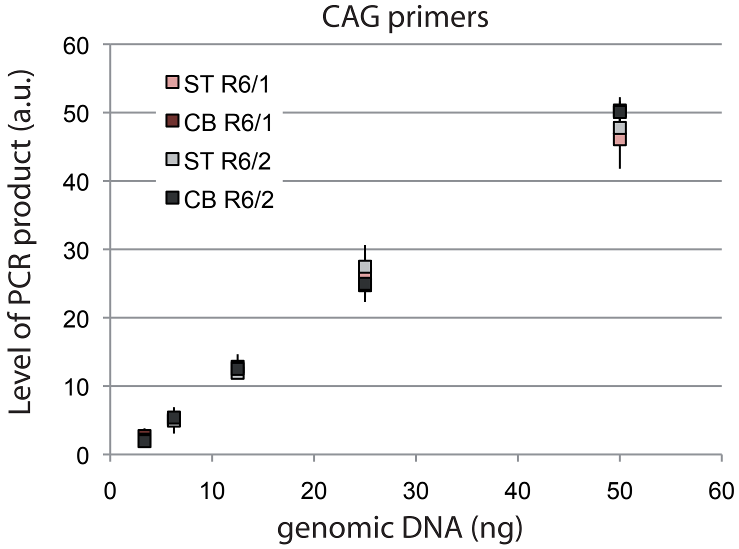

B

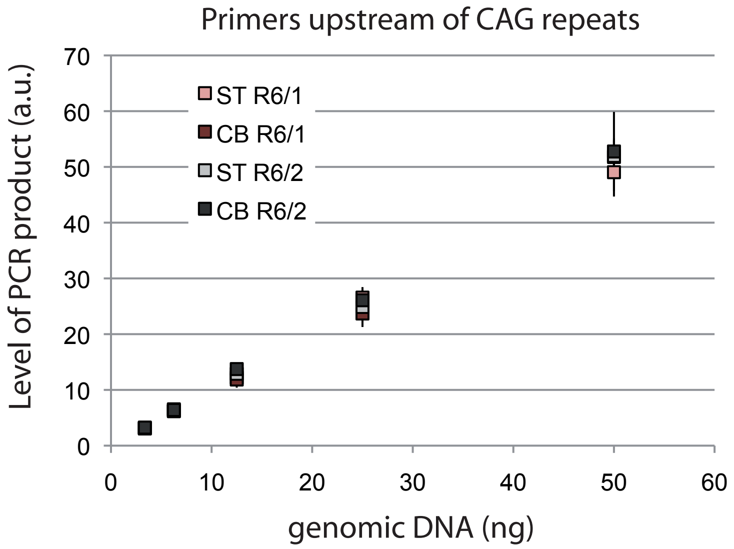

Fig. S4
